# Profiling and Identification of Omeprazole Metabolites in Mouse Brain and Plasma by Isotope Ratio-Monitoring Liquid Chromatography-Mass Spectrometric Method

**DOI:** 10.3390/life10070115

**Published:** 2020-07-19

**Authors:** Seok-Ho Shin, Yuri Park, Min-Ho Park, Jin-Ju Byeon, Byeong ill Lee, Jangmi Choi, Young G. Shin

**Affiliations:** College of Pharmacy and Institute of Drug Research and Development, Chungnam National University, Daejeon 34134, Korea; seokho.shin.cnu@gmail.com (S.-H.S.); yuri.park.cnu@gmail.com (Y.P.); minho.park.cnu@gmail.com (M.-H.P.); jinju.byeon.cnu@gmail.com (J.-J.B.); byungill.lee.cnu@gmail.com (B.i.L.); jangmi.choi.cnu@gmail.com (J.C.)

**Keywords:** omeprazole, proton-pump inhibitor, isotope ratio-monitoring, LC–QTOF–MS, metabolite identification, brain-to-plasma coefficient

## Abstract

Neuro–inflammation is known to be one of the pathogenesis for the degenerative central nervous system (CNS) disease. Recently various approaches for the treatment of brain diseases by controlling neuro-inflammation in the brain have been introduced. In this respect, there is a continuous demand for CNS drugs, which could be safer and more effective. Omeprazole, a well-known proton-pump inhibitor (PPI) is generally prescribed for the treatment of peptic ulcer. In addition to the anti-gastric acid secretion mechanism, recent studies showed that omeprazole or PPIs would likely have anti-inflammation effects in vitro and in vivo, but their effects on anti-inflammation in brain are still unknown. In this study, omeprazole and its metabolites in a mouse’s brain after various routes of administration have been explored by stable isotope ratio-patterning liquid chromatography–mass spectrometric method. First, a simple liquid chromatography–mass spectrometric (LC–MS) method was established for the quantification of omeprazole in mouse plasma and brain. After that, omeprazole and its stable isotope (D3–omeprazole) were concomitantly administered through various routes to mice in order to identify novel metabolites characteristically observed in the mouse brain and were analyzed using a different LC–MS method with information-dependent analysis (IDA) scan. With this unique approach, several new metabolites of omeprazole were identified by the mass difference between omeprazole and stable isotope in both brain and plasma samples. A total of seventeen metabolites were observed, and the observed metabolites were different from each administration route or each matrix (brain or plasma). The brain pharmacokinetic profiles and brain-to-plasma partition coefficient (Kp) were also evaluated in a satellite study. Overall, these results provide better insights to understand the CNS-related biological effects of omeprazole and its metabolites in vivo.

## 1. Introduction

Central nervous system (CNS) disease refers to a group of diseases encompassing abnormalities of the nervous system which include Parkinson’s disease (PD), Alzheimer’s disease (AD), multiple sclerosis (MS; myelin damage disease), neuropathic pain as well as schizophrenia, etc. [1,2,3,4]. In particular, neurodegenerative disease in the brain is known to be caused by abnormalities of neurons, and the basic research studies on the detailed mechanisms are at different levels of progress [3,4,5,6,7].

Recently, neuro-inflammation has been studied as the pathogenesis of this degenerative CNS disease, and various studies have been conducted to explore the mode of inflammation in the brain [8,9,10]. Although there have been numerous studies, the anti-inflammation mechanism in the brain is still under investigation. 

Omeprazole, which is classified as a proton-pump inhibitor (PPI), has indications for common peptic ulcers, such as gastro-esophageal reflux disease (GERD) and gastro-duodenal ulcers, and is used in combination with antibiotics to prevent germs and relapses in patients with *H. pylori*-positive peptic ulcers [11,12,13]. It is also prescribed for the treatment of peptic ulcers associated with non-steroidal anti-inflammatory drugs (NSAIDs) and for the prevention of peptic ulcers with sustained administration of NSAIDs. Recent studies have focused on the anti-inflammatory effect of omeprazole and other PPI drugs. The in vitro and in vivo studies of omeprazole revealed reduction of pro-inflammatory cytokines in the human microglial cells and the anti-inflammatory effect on CCI-induced neuropathic pain on the murine model [14,15,16]. Although explanations have been given for various anti-inflammatory mechanisms, to our best knowledge, no studies have focused on the omeprazole metabolites that might play a role in the brain. In order to investigate the roles of omeprazole metabolites in the brain, the first step would be to identify and characterize all relevant metabolites of omeprazole in the brain. Therefore, the object of this study is to identify the omeprazole metabolites observed in the mouse brain and compare them with the metabolites observed in plasma from in vivo mouse pharmacokinetics (PK) studies.

**Figure 1:**
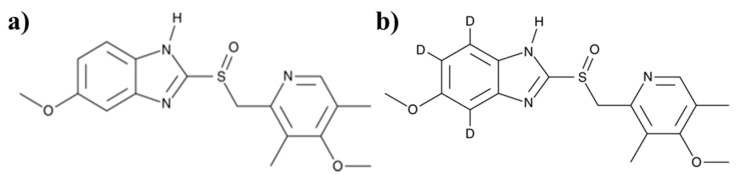
Structure of (**a**) omeprazole and its stable isotope (**b**) D3–omeprazole.

In terms of metabolites identification (MetID) from in vivo PK samples, one of the biggest challenges is to search for novel metabolites that do not follow the conventional rules of metabolic pathways (e.g., oxygenation, de-alkylation, hydrolysis, glucuronidation, sulfation, etc.) In this study, an approach using concomitant administration of omeprazole with a stable-isotope labeled omeprazole (D3–omeprazole) was used. The concomitant administration of stable isotope is a well-known method to identify characteristic metabolites of a certain compound [17,18,19]. Especially, the brain matrix contains various proteins, lipids and other endogenous materials, which can interfere the accurate detection of drug–related metabolites [20]. Therefore, omeprazole and its stable isotope D3–omeprazole (Figure 1) were concomitantly administered with 1:1 ratio (by the peak response in full scan mass spectra) to mice. In addition, various routes of administration including intraperitoneal (i.p.), intravenous (i.v.) and oral (p.o.) were applied to understand whether the metabolite profiles of omeprazole are different based on the administration route. The metabolites were then confirmed by monitoring the mass difference (3 daltons difference between omeprazole and D3–omeprazole) in full scan MS spectra in each sample for both brain and plasma samples. The brain/plasma pharmacokinetic profiles and brain-to-plasma partition coefficient (Kp) were also measured to determine the degree of distribution in the brain in a satellite study.

A simple LC–QTOF–MS assay was developed to quantify the concentrations of omeprazole from in vivo mouse plasma and brain samples to identify the brain distribution and pharmacokinetics by various dosing routes. The identification of metabolites from in vivo plasma/brain samples was also conducted using a LC–QTOF-MS assay containing information dependent analysis (IDA) method. To our knowledge, this is the first study to elucidate the omeprazole metabolites in mouse brain with different routes of administration. The results from this study should be helpful for future studies that reveal the effects of omeprazole metabolites in the brain neurology.

## 2. Materials and Methods

### 2.1. Reagents and Chemicals

Omeprazole was acquired from Tokyo chemical industry (Nihonbashi-honcho, Tokyo, Japan) while D3–omeprazole was acquired from Cayman chemical (Ann Arbor, MI 48108, USA). Verapamil was obtained from Merck & Sigma–Aldrich (Yong-in, Gyeonggi-Do, Korea). Dimethyl sulfoxide (DMSO), formic acid, methanol (MeOH), HPLC grade acetonitrile (ACN) and HPLC grade distilled water (DW) were all obtained from Duksan reagents (Ansan, Gyeonggi-Do, Korea).

### 2.2. Animals

All experimental protocols performed on mice were approved by the animal care institute from Chungnam National University (ptotocol no. CNU–01104). Male ICR mice (28~30 g) were purchased from the Samtako Biokorea Co. (Gyeonggi, Korea) and housed in groups of 4~5 per cage with free access to standard rodent chow (labdiet 5L79, Orientbio, Korea). All mice were kept for at least one week prior to starting the PK study and fasted 12 h prior to omeprazole/D3–omeprazole administration.

### 2.3. LC–QTOF–MS Condition 

The LC–QTOF-MS system was consisted of a chromatographic pump system (Shimadzu CBM–20A/LC–20AD, Shimadzu Scientific Instruments, Riverwood Dr, Columbia, SC, USA), an auto-sampler system (Eksigent CTC HTS PAL, Sciex, Redwood City, CA, USA), equipped with a mass spectrometer (TripleTOF^TM^ 5600, Sciex, Redwood City, CA, USA). Chromatographic separation was performed using reversed-phase C_18_ columns (Phenomenex^®^ Kinetex XB–C18 column; 2.1 × 50 mm for bioanalytical sample quantification and 2.1 × 100 mm for MetID). The HPLC mobile phase was comprised of distilled/deionized water containing 0.1% formic acid (phase A) and acetonitrile containing 0.1% formic acid (phase B) with a binary gradient program. The LC–gradient was optimized as follows: 3.2 min for the quantification (0–0.5 min, 5% B; 0.5–1.2 min, 5–95% B; 1.2–1.4 min, 95% B; 1.4–1.5 min, 95–5% B; and 1.5–3.2 min, 5% B with a flow rate of 0.4 mL/min) and 23 min for MetID (0–1 min, 5% B; 1–13 min, 5–40% B; 13–13.5 min, 40–45% B; 13.5–15.5 min, 45–90% B; 15.5–17.9 min, 90% B; 17.9–18 min, 90–5% B; and 18–23 min, 5% B with a flow rate of 0.3mL/min). In particular, the LC separation conditions for MetID studies were optimized where the peaks of metabolites were separated as much as possible. This was critical in searching for any relevant metabolites originated from omeprazole with unique isotope ratio 1:1.

The single reaction monitoring at high sensitivity option (SRMHS) in the product ion scan was used for the quantification of PK as well as MetID samples. The information-dependent analysis (IDA) method including high resolution TOF full scan and seven unique information-dependent product ion scans were performed for the MetID study. The ion spray voltage (ISVF) was set at 5500 V. The source gas (nebulizer [GS1, ion source gas1] and heater [GS2, ion source gas2]) was set at 50 psi, and the source temperature was set at 500 °C with the curtain gas (CUR) flow of 33 L/min. Other mass spectrometric conditions are summarized in Table 1 and Table 2.

### 2.4. Pharmacokinetics and Metabolite Identification

Omeprazole formulation was freshly prepared on the day of experiment. Mice were randomly distributed into three different groups (twelve mice per group; p.o. group, i.v. group and i.p. group) and received 10 mg/kg of omeprazole. Mice were sacrificed at 5, 15, 30 and 60 min (n = 3 for each time point). Blood was drawn into the heparinized tubes through cardiac puncture and was immediately centrifuged at 10,000 rpm for 5 min. The supernatant of the centrifuged samples was transferred to the clean tubes and stored at −80 °C until analysis. The whole brain was removed from the skull after systemic perfusion with phosphate buffered saline (PBS) and rinsed with PBS and was immediately homogenized in ice–cold PBS at a ratio of 1g brain tissue/4 mL ice–cold PBS. The homogenized samples were stored at −80 °C until usage.

A separate in vivo MetID study was conducted. A total of 50 mg/kg (1:1 ratio of omeprazole and D3–omeprazole) was given through each administration route (p.o., i.v. and i.p.). After administration, blood and brain tissue sampling was conducted in the same manner as the previous PK studies. The collected samples were stored at −80 °C until analysis.

### 2.5. Sample Preparation—In Vivo PK Samples

1. Plasma samples: Ten microliters of the mouse plasma samples were placed in cluster tubes. As a make-up solution, 4 μL of DMSO and 10 μL of blank brain homogenate were added. Hundred microliters of ACN containing internal standard (ISTD, verapamil) was added to each sample for extraction. The mixture was capped, gently shaken for approximately 1 min and then was centrifuged for 5 min at 10,000 rpm (4 °C). Following the centrifugation, 50 μL supernatant was transferred to a clean test tube and was diluted with 100 μL of distilled water. The resulting mixture was then transferred to an LC–vial and 10 μL was injected to the LC–QTOF–MS.
2. Brain samples: Ten microliters of the mouse brain homogenate were placed in cluster tubes. Four microliters of DMSO and 10 μL of blank plasma were added as a make-up solution. Hundred microliters of ACN containing internal standard (ISTD, verapamil) was added to each sample for extraction. The mixture was capped, gently shaken for approximately 1 min and then centrifuged for 5 min at 10,000 rpm (4 °C). Following the centrifugation, 50 μL supernatant was transferred to a clean test tube and was diluted with 100 μL of distilled water. The resulting mixture was then transferred to an LC-vial, and 10 μL was injected to the LC–QTOF–MS.
3. Standard (STD) and quality control (QC) samples: Ten microliters of the blank mouse plasma samples were placed in cluster tubes, and 10 μL of blank brain homogenate were added. Four microliters of STD samples (final concentrations of 3.02, 9.05, 27.2, 81.5, 244, 733, 2200 and 6670 ng/mL, respectively) and QC samples (low QC [15.0 ng/mL], medium QC [165 ng/mL] and high QC [1820 ng/mL], as final concentrations) were added to each cluster tube. The mixture was capped, gently shaken for approximately 1 min and then centrifuged for 5 min at 10,000 rpm (4 °C). Following the centrifugation, 50 μL supernatant was transferred to a clean test tube and was diluted with 100 μL of distilled water. The resulting mixture was then transferred to an LC–vial, and 10 μL was injected to the LC–QTOF–MS.

### 2.6. Sample Preparation—Metabolite Identification

The plasma samples obtained from the study dosed with 50 mg/kg were collected according to the Hamilton pooling method [21]. The pooled plasma sample (a total of 274 μL) was transferred to a clean tube, and 1 mL of ACN was added. The pretreated samples were centrifuged at 10,000 rpm for 5 min, and 1100 μL of the supernatant was evaporated to dryness under vacuum in a centrifugal evaporator (CVE–3110, Eyela, Tokyo, Japan) connected to cold trap (UT–1000, Eyela, Tokyo, Japan). The dried residue was re-constituted, shaken and centrifuged. After centrifugation, the supernatant was transferred to an LC–vial for analysis.

### 2.7. Software

Data acquisition and LC–QTOF-MS operation was conducted using Analyst^®^ TF Version 1.6 (Sciex). MultiQuant^®^ Version 2.1.1 (Sciex) was used for the peak integration of omeprazole for quantification. PeakView^®^ Version 2.2 and MetabolitePilot^TM^ Version 2.0.2 were used for the structural elucidation of omeprazole metabolites. Pharmacokinetic parameters were calculated in a non-compartmental analysis using WinNonlin^®^ version 8.0.0 (Certara, Princeton, NJ, USA).

## 3. Results

### 3.1. Method Development and Qualification

The optimum LC–QTOF–MS condition for quantification of omeprazole was evaluated in the positive ion mode using various combinations of mobile phase conditions and HPLC columns. The best condition was observed with DW/ACN (each containing 0.1% formic acid) as mobile phase and a Phenomenex^®^ Kinetex XB–C18 column (2.1 × 50 mm, 2.6 μm). The major ion of omeprazole during TOF full scan was the protonated [M + H]^+^ ion at *m/z* 346.1. Moreover, the most abundant product ion (*m/z* 198.1) was selected for the quantification using the SRMHS transition for omeprazole (346.1 → 198.1). The information-dependent analysis (IDA) method was also optimized for all metabolites derived from in vivo MetID studies. The same mobile phase condition was used for the method development, while a longer column (Phenomenex^®^ Kinetex XB–C18 column, 2.1 × 100 mm, 2.6 μm) with a different LC–gradient was used for the MetID sample analysis. 

The developed assay was well applied for the PK study with the calibration range of 3.02~6670 ng/mL (correlation coefficient ≥ 0.999, quadratic regression, weighted 1/concentration, curve equation: y = −2.08411e^−8^ x^2^ + 7.74056e^−4^ x + 6.90506e^−4^). Three levels of QC samples (low QC [15.0 ng/mL], medium QC [165 ng/mL] and high QC [1820 ng/mL], n = 3 each) were used for the determination of assay performance by assessing the precision (RSD (%)) and the mean accuracy (%). The repeated injection of LLOQ [3.02 ng/mL] was also performed to assess the assay performance. All samples met the acceptance criteria for this fit-for-purpose research study within ± 25% of the nominal value, and the results are summarized in Table 3.

### 3.2. Application

#### 3.2.1. Pharmacokinetic Study

**Figure 2:**
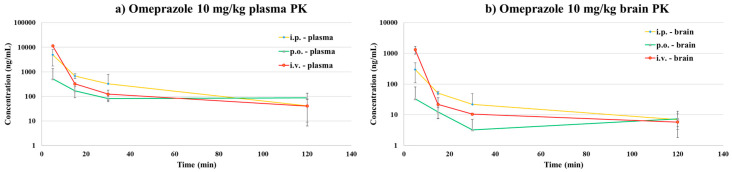
Time-concentration profiles of omeprazole from (**a**) plasma PK samples and (**b**) brain PK samples.

The developed LC–QTOF-MS method was successfully applied to determine the PK parameters after i.v., i.p. and p.o. administration at a dose level of 10 mg/kg each for omeprazole in mice. During the sample preparation of the PK samples, QC samples were included for the assurance of the bioanalytical run. All the PK samples were within the range of the qualified calibration range (3.02~6670 ng/mL). Final concentrations were calculated by considering the dilution factors (e.g., 4× and 2× of the concentrations obtained from the brain and plasma, respectively). Each PK parameters were measured by non-compartmental analysis using WinNonlin (version 8.0.0). The PK parameters and time-concentration profile of omeprazole are shown in Table 4 and Figure 2.

The i.v. PK study results showed that omeprazole has a moderate clearance (37.75 ± 5.05 mL/min/kg) in mice. The brain-to-plasma coefficient (Kp) was calculated to evaluate the efficiency of omeprazole passing through the brain [22,23]. The ratios between area under the curve (AUC_last_) values from brain and plasma were used for the calculation (Equation (1)) and as a result, the Kp value was 0.15 for i.v. administration of omeprazole.

Equation (1) brain to plasma partition coefficient:

(Kp) = AUC_brain_/AUC_plasma_ (1)

A value corresponding to the Kp value of a commercial CNS drug (Kp = 0.1~24 in mice) was obtained, and it was confirmed that omeprazole could penetrate the brain. The i.p. and p.o. PK profile also showed a similar profile for both brain and plasma samples. Although the Kp values obtained from these studies were smaller (0.068 and 0.064 for IP and PO, respectively) than those of the IV result, it was confirmed that omeprazole was able to penetrate the brain after both i.p. and p.o. administration. The average bioavailability of omeprazole was 24.06 and 5.31% for i.p. and p.o. administration routes, respectively.

#### 3.2.2. In Vivo Metabolite Identification

The metabolites of omeprazole were investigated using an LC–QTOF–MS assay. Simultaneous administration of the stable isotope labeled D3–omeprazole has been very helpful in the identification of drug-derived metabolites, especially in the brain samples. In this case, any metabolites having a mass difference of 3 daltons between omeprazole and its stable isotope D3–omeprazole were thoroughly searched as possible drug-related metabolites. Under the current experimental conditions, a total of seventeen metabolite peaks were observed from the in vivo MetID study, which includes 5–hydroxyomeprazole, omeprazole sulfone, etc. In addition, some metabolites (e.g., hydroxy glucuronide metabolite) have been newly identified for the first time in this study. 

**Figure 3:**
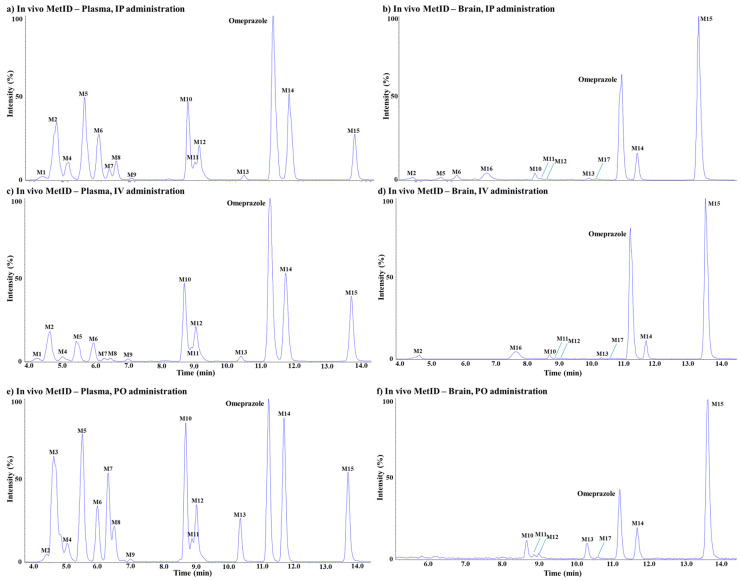
Extracted ion chromatogram of omeprazole MetID samples; (**a**) i.p. route (plasma); (**b**) i.v. route (plasma); (**c**) p.o. route (plasma); (**d**) i.p. route (brain); (**e**) i.v. route (brain); (**f**) p.o. route (brain).

**Figure 4:**
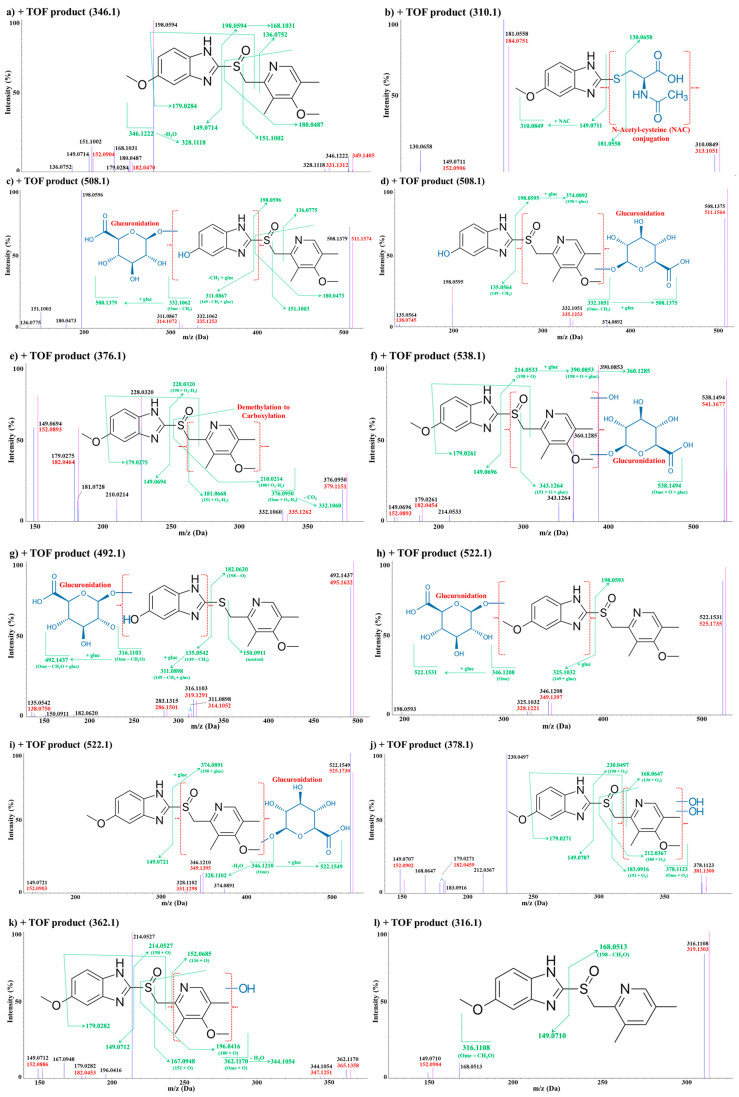

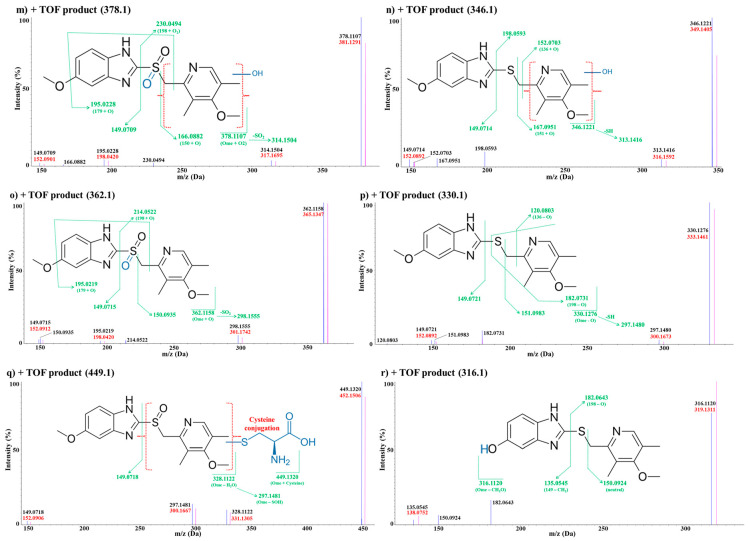
Identification of in vivo metabolites based on omeprazole. (**a**) TOF-MS/MS scan of omeprazole (*m/z* 346.1); (**b**) M1 (loss of C_9_H_11_NO_2_S followed by N-acetylcysteine conjugation, *m/z* 310.1); (**c**) M2 (demethylation followed by glucuronidation, *m/z* 508.1); (**d**) M3 (demethylation followed by glucuronidation, *m/z* 508.1); (**e**) M4 (demethylation to carboxylic acid, *m/z* 376.1); (**f**) M5 (oxidation followed by glucuronidation, *m/z* 538.1); (**g**) M6 (loss of CH_2_O followed by glucuronidation, *m/z* 538.1); (**h**) M7 (glucuronidation, *m/z* 522.1); (**i**) M8 (glucuronidation, *m/z* 522.1); (**j**) M9 (di-oxidation, *m/z* 378.1); (**k**) M10 (mono-oxidation, *m/z* 362.1); (**l**) M11 (loss of CH_2_O, *m/z* 316.1); (**m**) M12 (di-oxidation, *m/z* 378.1); (**n**) M13 (oxidation and de-oxidation, *m/z* 346.1); (**o**) M14 (mono-oxidation, *m/z* 362.1); (**p**) M15 (sulfoxide to thioether, *m/z* 330.1); (**q**) M16 (cysteine conjugation, *m/z* 449.1); (**r**) M17 (loss of CH_2_O, *m/z* 316.1).

**Figure 5:**
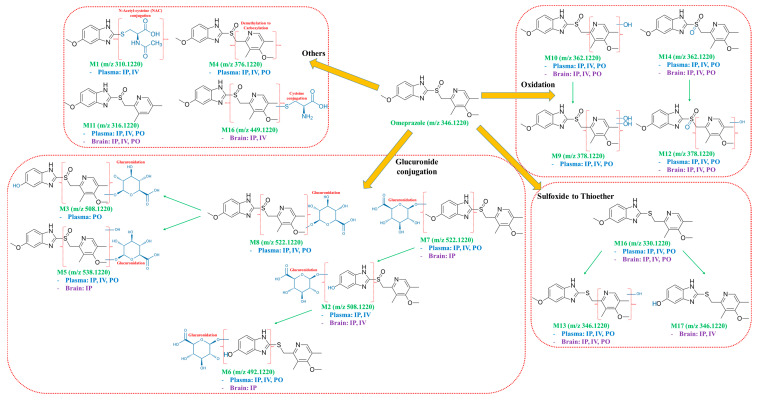
Metabolic pathways of omeprazole from plasma and brain samples.

Furthermore, the observed metabolites showed different ratios depending on the route of administration and the sample composition (plasma or brain) analyzed. Interestingly, the MetID results showed that some metabolites were found only in plasma, and some were found only in the brain. The different abundance of metabolites in different organs would be to some extent due to different levels of metabolic enzymes in each organ. The LC–QTOF-MS chromatographic separation of omeprazole and its metabolites in each administration route (i.v., i.p., p.o.)/matrix (plasma and brain) are shown in Figure 3. Each of the metabolites were analyzed using product ion scans (TOF-MS/MS) in positive ion mode to elucidate the structures (Figure 4), and the metabolic pathways of omeprazole are described in Figure 5.

##### Omeprazole

Omeprazole showed a molecular ion [M + H]^+^ at *m/z* 346. The TOF–MS/MS analysis of *m/z* 346 leads to the formation of fragment ions at *m/z* 328, 198, 180, 179, 151, 149, 136 and 121. The major fragment ion *m/z* 198 is formed by the neutral loss of 6–methoxy–1H–1,3–benzodiazole (C_8_H_8_N_2_O) from *m/z* 346. The subsequent loss of H_2_O from *m/z* 198 leads to the formation of *m/z* 180. The fragment ion *m/z* 149 is formed by the neutral loss of (4–methoxy–3,5–dimethylpyridine–2–yl)methanesulfinyl (C_9_H_11_NO_2_S) moiety from *m/z* 346. Other detailed fragmentation information and TOF–MS/MS data of omeprazole are shown in Figure 4a.

##### Metabolite M1

M1 showed a molecular ion [M + H]^+^ at *m/z* 310. TOF–MS/MS analysis of *m/z* 310 leads to the formation of fragment ions at *m/z* 181, 149 and 130. The unchanged fragment ion *m/z* 149 suggests that metabolism has occurred in (4–methoxy–3,5–dimethylpyridine–2–yl)methanesulfinyl moiety of omeprazole. The product ion at *m/z* 149, which is also a loss of 161 amu from the molecular ion of M1, suggests the presence of N–acetyl–cysteine (NAC) conjugation. The most significant fragment ion produced from M1 was the protonated mercaptobenzimidazole (*m/z* 181). In the case of fragment ion *m/z* 130, it was detected in both metabolites of omeprazole and its stable isotope D3–omeprazole, which means that this fragment ion was generated from NAC, not from the benzimidazole moiety. The accurate masses measured for all metabolites including M1 and the mass difference between the theoretical values and the observed ones are summarized in Table 5. Based on these results, M1 was suggested to be a NAC metabolite of the mercaptobenzimidazole of omeprazole. M1 was also observed in the plasma–IV route and plasma-IP route samples. The detailed fragmentation information and the TOF–MS/MS spectrum of M1 are shown in Figure 4b.

##### Metabolite M2

M2 showed a molecular ion [M + H]^+^ at *m/z* 508, which was 162 amu higher than the molecular ion of omeprazole, suggesting a demethylation followed by glucuronide conjugation was taking place in omeprazole. The TOF–MS/MS analysis of *m/z* 508 leads to the formation of fragment ions at *m/z* 332, 311, 198, 180, 151 and 136. The unchanged fragment ions at *m/z* 198, 180, 151 and 136 were all observed from both M2 and omeprazole/D3-omeprazole, suggesting that metabolism has likely occurred in 6-methoxy–1H–1,3–benzodiazole moiety of omeprazole. The fragment ion at *m/z* 332 is 14 amu less than the molecular ion of omeprazole and also 176 amu less than the molecular ion of M2 at *m/z* 508, suggesting that glucuronide (*m/z* 176) conjugation occurred in the demethylated form of omeprazole. M2 was observed in all samples except the brain–PO route sample. The detailed fragmentation information and the TOF–MS/MS spectrum of M2 are shown in Figure 4c.

##### Metabolite M3

M3 also showed a molecular ion [M + H]^+^ at *m/z* 508. However, the TOF-MS/MS analysis of M3 leads to the formation of fragment ions at *m/z* 374, 332, 198 and 135. The fragment ion of M3 at *m/z* 135 is 14 amu less than the fragment ion of omeprazole (*m/z* 149), suggesting that demethylation occurred in the 6–methoxy–1H–1,3–benzodiazole moiety of omeprazole. Similar to M2, a fragment ion at *m/z* 332 was observed (a loss of glucuronide by 176 amu from *m/z* 508), suggesting that glucuronidation *m/z* likely occurred in M3. However, unlike M2, a fragment ion at *m/z* 151 was not observed in M3. The unchanged fragment ion at *m/z* 198 and the de-glucuronide fragment ion at *m/z* 374 were observed from both M3 metabolites of omeprazole and its stable isotope D3–omeprazole, suggesting that metabolism occurred in the (4–methoxy–3,5–dimethylpyridine–2–yl) methanesulfinyl moiety. M3 was observed only from the plasma–PO route sample. The detailed fragmentation information and TOF–MS/MS spectrum are shown in Figure 4d.

##### Metabolites M4

M4 showed a molecular ion [M + H]^+^ at *m/z* 376, which was 30 amu higher than the molecular ion of omeprazole, suggesting a di–oxidation followed by reduction was likely taking place on omeprazole. The TOF–MS/MS analysis of *m/z* 376 leads to the formation of fragment ions at *m/z* 332, 228, 210, 181, 179 and 149. The unchanged fragment ions at *m/z* 179 and 149 suggest that no metabolic modification occurred in the 6–methoxy–1H–1,3–benzodiazole moiety of omeprazole. The fragment ions at *m/z* 228, 210 and 181 were 30 units higher than those of omeprazole fragments at *m/z* 198, 180 and 151. Moreover, these fragment ions were observed in both M4 metabolites of omeprazole and its stable isotope D3–omeprazole, suggesting that metabolism occurred in the 4–methoxy–3,5–dimethylpyridine–2–yl moiety of omeprazole. In particular, the neutral loss of 44 amu between *m/z* 332 and *m/z* 376 suggests that this M4 may contain carboxylic acid (*m/z* 376 – 44 (CO_2_) = *m/z* 332) in the structure. These results suggest that M4 was a modification of methyl group to carboxylic acid from the 4–methoxy–3,5–dimethylpyridine–2–yl moiety. M4 was observed in all plasma samples (all dosing route) but not from the brain samples. The detailed fragmentation information and TOF–MS/MS spectrum are shown in Figure 4e.

##### Metabolite M5

M5 showed a molecular ion [M + H]^+^ at *m/z* 538, which was 192 amu higher than the molecular ion of omeprazole, suggesting a mono–oxidation followed by glucuronide conjugation was taking place in omeprazole. The TOF–MS/MS analysis of *m/z* 538 leads to the formation of fragment ions at *m/z* 390, 360, 343, 214, 179 and 149. The unchanged fragment ions *m/z* 179 and 149 suggest that metabolism occurred in 4–methoxy–3,5-dimethylpyridin–2–yl moiety of omeprazole. The fragment ion at *m/z* 214 was 16 amu higher than *m/z* 198, suggesting that mono-oxidation also occurred. Fragment ions at *m/z* 390 and 343 were all 192 amu higher than the omeprazole fragments at *m/z* 198 and 151, suggesting a mono-oxidation followed by glucuronide conjugation occurred. These results suggest that M5 was a metabolite of glucuronidation and mono-oxidation of omeprazole. M5 was observed in all plasma samples (all dosing route) and brain–IP route sample. The detailed fragmentation information and TOF–MS/MS spectrum are shown in Figure 4f.

##### Metabolite M6

M6 showed a molecular ion [M + H]^+^ at *m/z* 492, which was 146 amu higher than the molecular ion of omeprazole, which appears to be a combination of de-methylation, de-oxygenation and glucuronide conjugation. The TOF–MS/MS analysis of *m/z* 492 leads to the formation of fragment ions at *m/z* 316, 283, 182, 150 and 135. Based on the fragment ion *m/z* 182, which was 16 units less than *m/z* 198, and the unchanged fragment ion *m/z* 150 observed in both M6 metabolites of omeprazole and its stable isotope D3–omeprazole, the de-oxygenation likely occurred in the sulfoxide moiety of omeprazole. The fragment ion at *m/z* 135 was 14 units less than *m/z* 149, which also confirmed the demethylation in M6. The combination of these two events resulted in a net loss of 30 units from omeprazole to form a fragment ion at *m/z* 316. These results suggest that M6 was a glucuronide conjugated metabolite to the O–desmethyl omeprazole sulfide. M6 was observed in all plasma samples (all dosing route) and brain–IP route sample. The detailed fragmentation information and TOF–MS/MS spectrum are shown in Figure 4g.

##### Metabolites M7

M7 showed a molecular ion [M + H]^+^ at *m/z* 522, which was 176 amu higher than the molecular ion of omeprazole, suggesting a glucuronide conjugation was taking place in omeprazole. The TOF–MS/MS analysis of *m/z* 522 led to the formation of fragment ions at *m/z* 346, 325 and 198. The unchanged fragment ion *m/z* 198 was observed in both M7 metabolites of omeprazole and its stable isotope D3–omeprazole, suggesting that metabolism occurred in 6–methoxy–1H–1,3–benzodiazole moiety of omeprazole. In addition, the fragment ion at *m/z* 325 was 176 amu higher than the fragment ion at *m/z* 149, suggesting that the glucuronide (*m/z* 176) conjugation site was the 6–methoxy–1H–1,3–benzodiazole moiety of omeprazole. M7 was observed in all plasma samples (all dosing routes) but was not observed from the brain samples. The detailed fragmentation information and TOF–MS/MS spectrum are shown in Figure 4h.

##### Metabolites M8

M8 also showed a molecular ion [M + H]^+^ at *m/z* 522. However, the TOF–MS/MS analysis of *m/z* 522 led to the different formation of fragment ions from M7 at *m/z* 374, 346, 328 and 149. The unchanged fragment ion at *m/z* 149 suggests that metabolism occurred in (4–methoxy–3,5–dimethylpyridine–2–yl)methanesulfinyl moiety of omeprazole. The fragment ion at *m/z* 374 was 176 amu higher than the fragment ion at *m/z* 198, suggesting that glucuronide (*m/z* 176) conjugation occurred in the (4–methoxy–3,5–dimethylpyridin–2–yl)methanesulfinyl moiety of omeprazole. M8 was observed in all plasma samples (all dosing route) but was not observed from the brain samples. The detailed fragmentation information and TOF–MS/MS spectrum are shown in Figure 4i.

##### Metabolites M9

M9 showed a molecular ion [M + H]^+^ at *m/z* 378, which was 32 amu higher than the molecular ion of omeprazole, suggesting a di-oxidation was taking place in omeprazole. The TOF–MS/MS analysis of *m/z* 378 led to the formation of fragment ions at *m/z* 230, 212, 183, 179, 168 and 149. The unchanged fragment ions at *m/z* 179 and 149 suggest that no metabolic modification occurred in the 6–methoxy–1H–1,3–benzodiazole moiety of omeprazole. The fragment ions at *m/z* 230, 212, 183 and 168 were all observed from both M9 metabolites of omeprazole and its stable isotope D3-omeprazole and were 32 amu higher than the omeprazole fragments at *m/z* 198, 151 and 136. These results suggest that M9 is a di–oxidation metabolite of omeprazole. M9 was observed in all plasma samples (all dosing routes) but was not observed from the brain samples. The detailed fragmentation information and TOF–MS/MS spectrum are shown in Figure 4j.

##### Metabolite M10

M10 showed a molecular ion [M + H]^+^ at *m/z* 362, which was 16 amu higher than the molecular ion of omeprazole, suggesting a mono-oxidation was taking place in omeprazole. The TOF–MS/MS analysis of *m/z* 362 led to the formation of fragment ions at *m/z* 344, 214, 196, 179, 167, 152 and 149. The unchanged fragment ions *m/z* 179 and 149 suggested that the 6–methoxy–1H–1,3–benzodiazole moiety of omeprazole was still intact. The fragment ions at *m/z* 214, 196, 167 and 152 were all observed from both M10 metabolites of omeprazole and its stable isotope D3–omeprazole and were 16 amu higher than the omeprazole fragments at *m/z* 198, 180, 151 and 136. These results suggest that M10 is a mono-oxidation metabolite of omeprazole. M10 was observed in all plasma samples (all dosing routes) and all brain samples (all dosing routes). The detailed fragmentation information and TOF–MS/MS spectrum are shown in Figure 4k.

##### Metabolite M11

M11 showed a molecular ion [M + H]^+^ at *m/z* 316, which was 30 amu less than the molecular ion of omeprazole, suggesting demethylation (-14) and deoxygenation (-16) were taking place in omeprazole. The TOF–MS/MS analysis of *m/z* 316 leads to the formation of fragment ions at *m/z* 168 and 149. The unchanged fragment ion *m/z* 149 suggested the 6–methoxy–1H–1,3–benzodiazole moiety of omeprazole was still intact. The fragment ion 168 was observed in both M11 metabolites of omeprazole and its stable isotope D3–omeprazole and was 30 amu less than the omeprazole fragment at *m/z* 198, suggesting a loss of hydroxymethylene (*m/z* 30) likely occurred in omeprazole. M11 was observed in all plasma samples (all dosing routes) and all brain samples (all dosing routes). The detailed fragmentation information and TOF-MS/MS data are shown in Figure 4l.

##### Metabolites M12

M12 showed a molecular ion [M + H]^+^ at *m/z* 378, which was 32 amu higher than the molecular ion of omeprazole, suggesting a di-oxidation was taking place on omeprazole, just as M7. However, the M12 fragment ion pattern was different from M7: *m/z* 314, 230, 195, 166 and 149. The unchanged fragment ion at *m/z* 149 suggested that no metabolic modification occurred in the 6–methoxy–1H–1,3–benzodiazole moiety of omeprazole. Instead, the fragment ions at *m/z* 195 and 166 were all 16 units higher than the fragment ions at *m/z* 179 and 150 from omeprazole, suggesting a mono-oxidation occurred on the other side functional group. In addition, the fragment ion at *m/z* 230 was 32 amu higher than *m/z* 198 suggesting a di-oxidation occurred in the (4–methoxy–3,5–dimethylpyridine–2–yl)methanesulfinyl moiety of omeprazole. The fragment ion at *m/z* 314 was 64 amu less than the molecular ion of M12, suggesting a neutral loss of SO_2_. These results suggest that sulfone was formed in the sulfoxide moiety, and an additional hydroxylation occurred in omeprazole. M12 was observed in all plasma samples (all dosing routes) and all brain samples (all dosing routes). The detailed fragmentation information and TOF–MS/MS data are shown in Figure 4m.

##### Metabolite M13

M13 showed a same molecular ion [M + H]^+^ at *m/z* 346 as omeprazole. However, the fragment ion pattern was quite different from omeprazole: *m/z* 313, 198, 167, 152 and 149. The unchanged fragment ions *m/z* 149 and 198 were observed in M13, but two fragment ions (*m/z* 167, 152) that were not identified from omeprazole were observed from M13. The fragment ions at *m/z* 167 and 152 were 16 amu higher than those at *m/z* 151 and 136, suggesting that hydroxylation occurred in the 4–methoxy–3,5–dimethylpyridine structure of omeprazole. The fragment ions at *m/z* 198 and 167 were both observed in the M13 metabolites of omeprazole and its stable isotope D3–omeprazole, suggesting that both the reduction and oxidation occurred in the (4–methoxy–3,5-dimethylpyridin–2–yl)methanesulfinyl moiety of omeprazole. These results suggested that M13 was a metabolite with sulfoxide reduction to thioether and mono–oxidation from omeprazole. M13 was observed in all plasma samples (all dosing routes) and all brain samples (all dosing routes). The detailed fragmentation information and TOF–MS/MS data are shown in Figure 4n.

##### Metabolite M14

M14 showed a molecular ion [M + H]^+^ at *m/z* 362, which was 16 amu higher than the molecular ion of omeprazole. However, unlike M10, the fragmentation pattern was different from M10 as follows: *m/z* 298, 214, 195, 150 and 149. The fragment ions at *m/z* 214 and 195 were 16 units higher than the omeprazole fragments *m/z* 198 and 179, suggesting an addition of oxygen occurred in omeprazole. The unchanged fragment ions at *m/z* 150 and 149 also suggested that the modification did not occur in either 6–methoxy–1H–1,3–benzodiazole moiety or 4–methoxy–2,3,5–trimethylpyridine moiety of omeprazole. The unique fragment ion at *m/z* 298 was 64 amu less than the molecular ion of M14, which implies a neutral loss of SO_2_. These results suggested that the modification was mono-oxidation, and it occurred in the sulfoxide moiety to form omeprazole sulfone. M14 was observed in all plasma samples (all dosing routes) and all brain samples (all dosing routes). The detailed fragmentation information and TOF-MS/MS data are shown in Figure 4o.

##### Metabolite M15

M15 showed a molecular ion [M + H]^+^ at *m/z* 330, which was 16 amu less than the molecular ion of omeprazole, suggesting a loss of oxygen from omeprazole. The TOF–MS/MS analysis of *m/z* 330 led to the formation of fragment ions at *m/z* 297, 182, 151, 149 and 120. The unchanged fragment ions at *m/z* 151 and 149 suggests that the modification did not occur either in 6–methoxy–1H–1,3–benzodiazole moiety or in 4–methoxy–2,3,5–trimethylpyridine moiety of omeprazole. The fragment ions *m/z* 182 and 120 were all observed from both M15 metabolites of omeprazole and its stable isotope D3–omeprazole and were 16 units less than fragment ions *m/z* 198 and 136. These results suggest that a loss of oxygen occurred from the sulfoxide moiety to form omeprazole sulfide. M15 was observed in all plasma samples (all dosing routes) and all brain samples (all dosing routes). The detailed fragmentation information and TOF-MS/MS data are shown in Figure 4p.

##### Metabolite M16

M16 showed a molecular ion [M + H]^+^ at *m/z* 449, which was 103 amu higher than the molecular ion of omeprazole, suggesting a cysteine conjugation occurred. The TOF–MS/MS analysis of *m/z* 449 led to the formation of fragment ions at 328, 297 and 149. All of these fragments showed 3 Da of mass difference in the metabolites of omeprazole and D3–omeprazole. The unchanged fragment ion 149 suggests that the modification did not occur in 6–methoxy–1H–1,3–benzodiazole moiety of omeprazole. The fragment ions at *m/z* 328 and 297 was 18 and 49 amu less than the corresponding ions from omeprazole, which implied a loss of H_2_O and SOH from omeprazole, respectively. These results suggest that M15 is a cysteine conjugation metabolite of omeprazole. M16 was observed only from the brain-IP route and brain-IV route. The detailed fragmentation information and TOF–MS/MS data are shown in Figure 4q.

##### Metabolite M17

M17 showed a molecular ion [M + H]^+^ at *m/z* 316, which was 30 amu less than the molecular ion of omeprazole, just as M11. However, its fragment ion pattern was different from M11: *m/z* 182, 150 and 135. Fragment ion at *m/z* 182 was 16 units less than the ion at *m/z* 198 of omeprazole, and the unchanged fragment ion at *m/z* 150 suggested the loss of oxygen occurred in the sulfoxide of omeprazole. The fragment ion *m/z* 135 was 14 units less than *m/z* 149 of omeprazole, suggesting a demethylation was also occurred in the 6–methoxy–1H–1,3–benzodiazole moiety of omeprazole. These results suggest that M17 was a demethylation and sulfoxide to thioether metabolite of omeprazole. M17 was observed only from the brain–IP route and brain–IV route. The detailed fragmentation information and TOF–MS/MS data are shown in Figure 4r.

## 4. Discussion and Conclusions

Omeprazole is classified as PPI, and its indications are for the treatment of common peptic ulcers such as GERD or NSAID related ulcers [11,12,13]. In addition to the previously known indications, research on anti-inflammatory mechanisms of omeprazole and other PPI drugs has recently been carried out [14,15,16]. In particular, the possibility of application to the underlying inflammatory response of brain diseases has been discussed [8,9,10]. Although various anti-inflammatory mechanisms have been proposed, to our best knowledge, most studies were conducted from the parent drug perspectives, and no studies have ever focused on the effect of metabolites of omeprazole possibly presented in the brain so far. With the view that metabolites also exhibit anti-inflammatory effect as well as parent drugs [24,25,26], we set up a hypothesis that some omeprazole metabolites observed in the brain might play a role regarding an anti-inflammatory effect. Therefore, the object of this study was to identify any possible omeprazole metabolites that can penetrate the brain through an in vivo mouse study as the first step.

Since the plasma-to-brain coefficient of distribution (Kp) value is the most widely used in vivo parameter for the assessment of drug penetration in the CNS system, we first measured the brain/plasma ratio of omeprazole to determine the degree of distribution in the brain [24,25]. Although there are some study results regarding the Kp, it was evaluated by the i.v. administration route only. Omeprazole is normally administered by oral (p.o.) route in human, and therefore, we also have conducted a study to determine the Kp by p.o. administration. In addition, we were also interested in exploring the other administration route, i.p., because several anti-inflammatory studies were conducted by i.p. administration [27,28], which is an useful way to demonstrate first-pass effect by liver without causing gastric acid stability issue. After p.o. administration, the Cmax in the brain was observed at a similar time point to the Cmax in plasma, indicating that omeprazole was very rapidly distributed to the brain with no/little lag time. The Kp was calculated to evaluate the efficiency of omeprazole passing through the brain. The ratios between area under the curve (AUC_last_) values from brain and plasma were used for calculating Equation (1). As a result, the Kp value was 0.15 for IV administration of omeprazole, the same value as in the existing literature [29,30]. Considering the Kp values of a commercial CNS drug (Kp = 0.1~24 in mice), the Kp of omeprazole appeared to have poor brain penetration [31,32,33]. The i.p. and p.o. administration results showed lower Kp values (0.0681 and 0.0643, respectively) and obviously the Kp differences between administration routes would be likely due to either metabolism or limited gastrointestinal penetration depending on the administration routes [34,35]. Particularly, due to the nature of decomposition by gastric acid and metabolism by metabolic enzymes [36,37], the route of administration may play a significant role in terms of BBB penetration of omeprazole and its metabolites [38]. To investigate this, in vivo MetID was conducted to explore all possible omeprazole metabolites in plasma as well as in brain by different routes of administration.

Some omeprazole metabolite studies have been conducted with in vitro/in vivo samples from various species [39,40,41,42] and in the environmental wastewaters [43]. However, no/little studies have been conducted to deal with the effects of omeprazole metabolites in relation to anti-inflammatory responses in CNS diseases, and therefore, the research on the metabolites in addition to the parent drug in the brain would be critical to better understand the mode of anti-inflammatory actions in vivo. Accurate mass measurement is an essential process to elucidate the elemental composition and structural information in the identification of metabolites. Especially, the usage of either radio-labeled or stable-isotope labeled compound would be helpful for the identification of novel metabolites [17,18,19]. In this study, we administered omeprazole and its stable isotope D3–omeprazole concomitantly in a dose level of 50 mg/kg (1:1 ratio of omeprazole and D3–omeprazole mixture) to mice and compared the metabolite production according to the route of administration in the mouse plasma and brain. With this unique isotope ratio monitoring approach, we were able to trace truly drug–related metabolites of omeprazole in mouse brain and plasma very efficiently.

A total of seventeen metabolites were identified, and there were differences in terms of metabolites observed depending on the route of administration and the matrix (brain or plasma) analyzed. Several metabolites previously identified were also confirmed in this study (e.g., omeprazole sulfone, omeprazole sulfide, hydroxyl–omeprazole, etc.) [39,40,41,42,43], but unreported metabolites (M1~M6, M16) were also newly identified from this study. Due to the absence of specific metabolic enzymes in rodents, some metabolites (e.g., 5-o-desmethylomeprazole) reported in humans were not identified in this study [42,44]. Interestingly, the ratios of major metabolites found in plasma and brain were different. The major metabolites observed in the plasma samples were related to the metabolic pathways of “oxidation + glucuronidation”, “mono–oxidation” and “sulfoxide to thioether”. On the other hand, the major metabolite observed in the mouse brain appeared to be more related to the “sulfoxide to thioether” metabolic pathway. It is unclear whether the different abundance of metabolic enzymes between liver and brain or the different BBB penetration capability of metabolites played a role in these different metabolic profiles between plasma and brain. Nevertheless, it is an interesting result and would be able to give us some clues to better understand the CNS-related biological activity in the brain.

It is also known that the thioether moiety has a higher binding capacity with a receptor presented in the brain than the sulfoxide or sulfone moiety, and this might be the reason why the relative abundance of omeprazole sulfide was higher in the brain [45]. Thioether derivatives are also known to possess anti-inflammatory effects, and therefore, the anti-inflammatory effects of omeprazole in the brain studied in recent researches may be to some extent due to the distribution of these thioether-type metabolites in the brain [46,47,48]. It is worth noting that the cysteine conjugation metabolite was only observed in the brain tissues (from the i.v. and i.p. administration study), and it might be related to active transporters, which can preferably influx compounds with the cysteine moiety. This is in line with the results of increased brain uptake of cysteine conjugated substances in the literatures [49,50].

In conclusion, we have developed a sensitive and simple LC–QTOF–MS method linked with isotope-ratio monitoring to quantify and to identify the omeprazole and omeprazole metabolites observed in the brain and plasma from three different routes of administrations in vivo for the first time. Particularly, we found different levels of metabolites in brain, which could be helpful to explain the effect about the neuro-inflammation, and further studies would be warranted to understand the in-depth mechanism of neuro-inflammation by these metabolites.

## Figures and Tables

**Table 1:** The mass spectrometric conditions for quantification.

Scan Mode	TOF-MS Scan	Product Ion Scan (SRMHS)	
		Omeprazole	Verapamil (ISTD)
**Mass range**	*m/z* 100–500	*m/z* 155–250	*m/z* 100–500
**Parent ion**	-	*m/z* 346.1	*m/z* 455.3
**Product ion**	-	*m/z* 198.1	*m/z* 165.1
**DP**	80 V	80 V	80 V
**CE**	7 V	15 V	33 V
**Accumulation time**	0.2 s	0.1 s	0.1 s

**Table 2:** The mass spectrometric conditions for metabolites identification (MetID).

Scan Mode	TOF-MS Scan	Product Ion Scan (IDA)
**Mass range**	*m/z* 100–1000	*m/z* 50–1000
**DP**	80 V	80 V
**CE**	7 V	18 V
**Accumulation time**	0.2 s	0.08 s
**Number of information dependent scans**	-	7 scans

**Table 3:** The intra/inter-run assays and repeated injection results for omeprazole.

**Intra-Run Assay**					
**Run**	**Nominal Concentration (ng/mL)**	**Calculated Concentration (ng/mL)**	**Mean Accuracy (%)**	**Precision (% CV)**	**n**
Run 1	QC low (15.02)	16.9	112.3	10.6%	3
	QC medium (165.29)	185.2	112.0	4.7%	
	QC high (1818.18)	1972.4	108.5	10.9%	
Run 2	QC low (15.02)	16.3	108.8	0.9%	3
	QC medium (165.29)	177.2	107.2	5.6%	
	QC high (1818.18)	1830.9	100.7	3.1%	
Run 3	QC low (15.02)	14.8	98.6	1.6%	3
	QC medium (165.29)	170.0	102.9	0.8%	
	QC high (1818.18)	1790.7	98.5	6.9%	
**Inter-Run Assay (Run 1~3)**					
**Nominal QC Concentration (ng/mL)**		**Calculated Concentration (ng/mL)**	**Mean Accuracy (%)**	**Precision (% CV)**	**n**
QC low (15.02)		14.1	94.0	10.3%	9
QC medium (165.29)		159.9	96.8	5.1%	
QC high (1818.18)		1710.4	94.1	3.1%	
**Repeat injection of LLOQ**					
**Nominal Concentration (ng/mL)**		**Calculated Concentration (ng/mL)**	**Mean Accuracy (%)**	**Precision (% CV)**	**n**
3.02		2.9	95.4	13.3%	6

**Table 4:** Pharmacokinetic parameters of omeprazole from i.v./p.o./i.p. pharmacokinetics (PK) study.

PK Parameters of Omeprazole.								
PK Study	Dose (mg/kg)	T_1/2_ (min)	C_max_ (ng/mL)	AUC_last_ (min·ng/mL)	Vd (mL/kg)	CL (mL/min/kg)	Brain/Plasma Ratio (%)	BA (%)
i.p.-brain	10	51.31	300.61	4365.73	-	-	6.81%	24.06%
i.p.-plasma		28.81	4885.82	64,115.37	-	-		
p.o.-brain	10	44.58	37.81	909.27	-	-	6.43%	5.31%
p.o.-plasma		54.76	610.15	14,149.52	-	-		
i.v.-brain	10	48.24	11,233.77	39,105.36	17,793.24	254.76	14.68%	-
i.v.-plasma		27.39	67,631.18	266,451.92	1491.70	37.75		

**Table 5:** In vivo omeprazole MetID results in various administration routes and study matrices.

In Vivo MetID Result of Omeprazole												
**Peak ID**	Name	Formula (H_3_ and D_3_)	*m/z*	Error (ppm)	Nominal Mass Change (Da)	RT (min)	A	B	C	D	E	F
Parent	Omeprazole [M + H]+	C_17_H_19_N_3_O_3_S	346.1220	0.6	-	11.250	O	O	O	O	O	O
		D_3_C_17_H_16_N_3_O_3_S	349.1408	−0.9								
M1	Loss of C9H11NO2S followed by N-acetylcysteine conjugation [M + H]+	C_13_H_15_N_3_O_4_S	310.0856	−2.3	−36	4.332	O	O	-	-	-	-
		D_3_C_13_H_12_N_3_O_4_S	313.1044	2.1								
M2	Demethylation followed by glucuronide conjugation [M + H]+	C_22_H_25_N_3_O_9_S	508.1384	−1	+162	4.420	O	O	O	O	O	-
		D_3_C_22_H_22_N_3_O_9_S	511.1573	0.3								
M3	Demethylation followed by glucuronide conjugation [M + H]+	C_22_H_25_N_3_O_9_S	508.1384	−1.8	+162	4.657	-	-	O	-	-	-
		D_3_C_22_H_22_N_3_O_9_S	511.1573	−1.7								
M4	Demethylation to carboxylic acid [M + H]+	C_17_H_17_N_3_O_5_S	376.0962	−3.1	+30	5.086	O	O	O	-	-	-
		D_3_C_17_H_14_N_3_O_5_S	379.1150	0.3								
M5	Oxidation followed by glucuronide conjugation [M + H]+	C_23_H_27_N_3_O_10_S	538.1490	0.8	+192	5.586	O	O	O	O	-	-
		D_3_C_23_H_24_N_3_O_10_S	541.1678	−0.2								
M6	Loss of hydroxymethylene followed by glucuronide conjugation [M + H]+	C_22_H_25_N_3_O_8_S	492.1435	0.4	+146	6.013	O	O	O	O	-	-
		D_3_C_22_H_22_N_3_O_8_S	495.1623	1.7								
M7	Glucuronide conjugation [M + H]+	C_23_H_27_N_3_O_9_S	522.1541	−1.9	+176	6.333	O	O	O	-	-	-
		D_3_C_23_H_24_N_3_O_9_S	525.1729	1.1								
M8	Glucuronide conjugation [M + H]+	C_23_H_27_N_3_O_9_S	522.1541	1.6	+176	6.533	O	O	O	-	-	-
		D_3_C_23_H_24_N_3_O_9_S	525.1729	0.2								
M9	Di-oxidation [M + H]+	C_17_H_19_N_3_O_5_S	378.1118	1.3	+32	6.986	O	O	O	-	-	-
		D_3_C_17_H_16_N_3_O_5_S	381.1307	−1.7								
M10	Mono-oxidation [M + H]+	C_17_H_19_N_3_O_4_S	362.1169	0.3	+16	8.679	O	O	O	O	O	O
		D_3_C_17_H_16_N_3_O_4_S	365.1357	0.2								
M11	Loss of hydroxymethylene [M + H]+	C_16_H_17_N_3_O_2_S	316.1114	−2.0	−30	8.878	O	O	O	O	O	O
		D_3_C_16_H_14_N_3_O_2_S	319.1303	0.1								
M12	Di-oxidation [M + H]+	C_17_H_19_N_3_O_5_S	378.1118	−3.0	+32	9.016	O	O	O	O	O	O
		D_3_C_17_H_16_N_3_O_5_S	381.1307	−2.5								
M13	Oxidation and Deoxidation [M + H]+	C_17_H_19_N_3_O_3_S	346.1220	0.3	-	10.356	O	O	O	O	O	O
		D_3_C_17_H_16_N_3_O_3_S	349.1408	−0.9								
M14	Mono-oxidation [M + H]+	C_17_H_19_N_3_O_4_S	362.1169	−3.0	+16	11.710	O	O	O	O	O	O
		D_3_C_17_H_16_N_3_O_4_S	365.1357	−2.8								
M15	Sulfoxide to thioether [M + H]+	C_17_H_19_N_3_O_2_S	330.1271	1.6	−16	13.656	O	O	O	O	O	O
		D_3_C_17_H_16_N_3_O_2_S	333.1459	0.6								
M16	Cysteine conjugation [M + H]+	C_20_H_24_N_4_O_4_S_2_	449.1312	1.8	+103	7.235	-	-	-	O	O	-
		D_3_C_20_H_21_N_4_O_4_S_2_	452.1500	1.3								
M17	Loss of hydroxymethylene [M + H]+	C_16_H_17_N_3_O_2_S	316.1114	1.8	−30	10.489	-	-	-	O	O	-
		D_3_C_16_H_14_N_3_O_2_S	319.1303	2.6								

A, i.p. route (plasma); B, i.v. route (plasma); C, p.o. route (plasma); D, i.p. route (brain); E, i.v. route (brain); F, p.o. route (brain); O, found; -, not found.
